# The Prefoldin Bud27 Mediates the Assembly of the Eukaryotic RNA Polymerases in an Rpb5-Dependent Manner

**DOI:** 10.1371/journal.pgen.1003297

**Published:** 2013-02-14

**Authors:** María Carmen Mirón-García, Ana Isabel Garrido-Godino, Varinia García-Molinero, Francisco Hernández-Torres, Susana Rodríguez-Navarro, Francisco Navarro

**Affiliations:** 1 Departamento de Biología Experimental, Facultad de Ciencias Experimentales, Universidad de Jaén, Jaén, Spain; 2 Centro de Investigación Príncipe Felipe (CIPF), Gene Expression Coupled with RNA Transport Laboratory, Valencia, Spain; The University of North Carolina at Chapel Hill, United States of America

## Abstract

The unconventional prefoldin URI/RMP, in humans, and its orthologue in yeast, Bud27, have been proposed to participate in the biogenesis of the RNA polymerases. However, this role of Bud27 has not been confirmed and is poorly elucidated. Our data help clarify the mechanisms governing biogenesis of the three eukaryotic RNA pols. We show evidence that Bud27 is the first example of a protein that participates in the biogenesis of the three eukaryotic RNA polymerases and the first example of a protein modulating their assembly instead of their nuclear transport. In addition we demonstrate that the role of Bud27 in RNA pols biogenesis depends on Rpb5. In fact, lack of *BUD27* affects growth and leads to a substantial accumulation of the three RNA polymerases in the cytoplasm, defects offset by the overexpression of *RPB5*. Supporting this, our data demonstrate that the lack of Bud27 affects the correct assembly of Rpb5 and Rpb6 to the three RNA polymerases, suggesting that this process occurs in the cytoplasm and is a required step prior to nuclear import. Also, our data support the view that Rpb5 and Rpb6 assemble somewhat later than the rest of the complexes. Furthermore, Bud27 Rpb5-binding but not PFD-binding domain is necessary for RNA polymerases biogenesis. In agreement, we also demonstrate genetic interactions between *BUD27*, *RPB5*, and *RPB6*. Bud27 shuttles between the nucleus and the cytoplasm in an Xpo1-independent manner, and also independently of microtubule polarization and possibly independently of its association with the RNA pols. Our data also suggest that the role of Bud27 in RNA pols biogenesis is independent of the chaperone prefoldin (PFD) complex and of Iwr1. Finally, the role of URI seems to be conserved in humans, suggesting conserved mechanisms in RNA pols biogenesis.

## Author Summary

The mechanisms governing the assembly and the transport of the three eukaryotic RNA polymerases to the nucleus are in discussion. Interesting papers have demonstrated the participation of some proteins in the assembly of the nuclear RNA polymerases and in their transport to the nucleus, but the mechanisms involved are poorly understood. Our data help clarify the mechanisms governing biogenesis of the three eukaryotic RNA pols and demonstrate that the prefoldin Bud27 of *Saccharomyces cerevisiae* mediates the correct assembly of the three complexes prior to their translocation to the nucleus, in a process which is dependent on Rpb5. In addition, our data support the view that, during the assembly of the RNA pols, Rpb5 and Rpb6 assemble rather late compared to the rest of the complexes. Furthermore, this role of Bud27 seems to be specific, as it is not extended to other prefoldin members. Finally, the role of Bud27 seems to be conserved in humans, suggesting conserved mechanisms in RNA pols biogenesis.

## Introduction

Eukaryotic RNA polymerases are a family of multimeric enzymes, RNA pol I, II, and III, responsible for the specific synthesis of different RNAs. RNA pol I is specialized in the synthesis of the pre-rRNA precursor of the three largest rRNA and typically account for about 75% of the entire transcription output in fast-growing yeast cells. RNA pol III transcribes mostly tRNAs and 5S rRNA, together with several short non-translated RNAs, while transcription corresponds to about 15% of the total RNA. RNA pol II, the enzyme that produces all mRNAs and many non-coding ones, transcribes most of the nuclear genome but nevertheless contributes to less than 10% of total RNA in growing cells. RNA pol I, II, and III are composed of 14, 12, and 17 subunits respectively, with a catalytic core formed by the two largest subunits highly conserved through evolution and five common subunits to the three enzymes [1]–[3]. Despite intensive studies concerning the structure and the transcriptional regulation of the three RNA polymerases [4], [5], little is known about the mechanisms governing their assembly and their nuclear import.

Noteworthy findings in both human and yeast demonstrate the participation of different proteins in the transport of the RNA pol II to the nucleus, Iwr1 and Npa3 in yeast, and GPN1 (RPAP4) and GPN3 in humans [6]–[10]. It has also been suggested that RPAP2 plays a role in import on the basis that it is cytoplasmic, binds fully assembled enzyme and shuttles in a CRM1-dependent manner [11]. However, no data concerning proteins involved in the nuclear transport of the RNA pol I or III are available. In addition, proteomic analysis in humans cells seek to decipher the mechanisms of RNA pol II biogenesis and assembly identifying a number of polymerase-associated factors. Among these, HSP90 and its R2TP/Prefoldin-like chaperone, including hSpagh (RPAP3), are clearly involved in these processes [8], [12].

In humans, R2TP/Prefoldin-like complex contains Rpb5, a common subunit to the three eukaryotic RNA polymerases [2], as well as the unconventional prefoldin Rpb5 interactor (URI/RMP), a member of the prefoldin (PFD) family of ATP-independent molecular chaperones [8],[13]. URI physically binds Rpb5, other nuclear proteins involved in transcription, including the general transcription factor TFIIF [14]–[16] and components of the Paf-1 complex that promotes RNA pol II CTD phosphorylation and histone modification during transcription elongation [17]. Notably, its yeast homologue Bud27 also binds Rpb5 [18]. URI was originally characterized in human and yeast cells as regulator of gene expression controlled by TOR (for target of Rapamycin) pathway [18]. Furthermore, URI has been linked to translation initiation [19], transcription regulation, chromatin stability or DNA damage response [13], [20]. URI is located mainly in the cytoplasm, although nuclear and perinuclear localization has also been observed in different organisms [20]–[22]. However, in *Saccharomyces cerevisiae*, only a cytoplasmic localization has been detected [19]. In addition, URI is believed to function as a scaffold protein able to assemble additional members of PFD family (through its PFD- and Rpb5-binding domains) in both human and yeast [13], [23] and different authors have proposed a role in the cytoplasmic assembly of the human RNA pol II [8], [12], [13].

Despite that some experiments, using immunoprecipitation and mass-spectrometry, have shown that URI interacts with RNA polymerases and other intermediary subcomplexes involved in RNA pol II cytoplasmic assembly, very little is known about the role of URI in RNA polymerases biogenesis. In this report, we investigate the role of the URI yeast homologue Bud27 in RNA polymerases assembly and its relationship with Rpb5. Bud27 interacts with different subunits of the three RNA polymerases, with the common subunit Rpb5 and Rpb10, as well as with a component of the prefoldin-like complex, Yke2 (Pfd6). Deletion of *BUD27* leads to a substantial accumulation of the three RNA polymerases in the cytoplasm, a defect offset by the overexpression of *RPB5*. Supporting this, our data demonstrate that the lack of Bud27 affects the correct assembly of Rpb5 and Rpb6 to the three RNA polymerases, suggesting that this process occurs in the cytoplasm and is a required step prior to nuclear import. As previously proposed in human [12], Rpb6 appears to assemble rather late after Rpb5 assembly. In agreement, we observed a genetic interaction between *BUD27*, *RPB5* and *RPB6*. In addition, Bud27 PFD- or Rpb5-binding domains are not necessary for RNA polymerases biogenesis. Finally, our data demonstrate that Bud27 shuttles between the cytoplasm and nucleus, as shown by the deletion of the NES domain, contrary to what has been previously described, a mechanism that is independent of the Xpo1-mediated pathway. URI silencing suggests that similar role for this protein accounts in humans.

## Results

### Bud27 interacts physically with the three eukaryotic RNA polymerases

**Figure 1:**
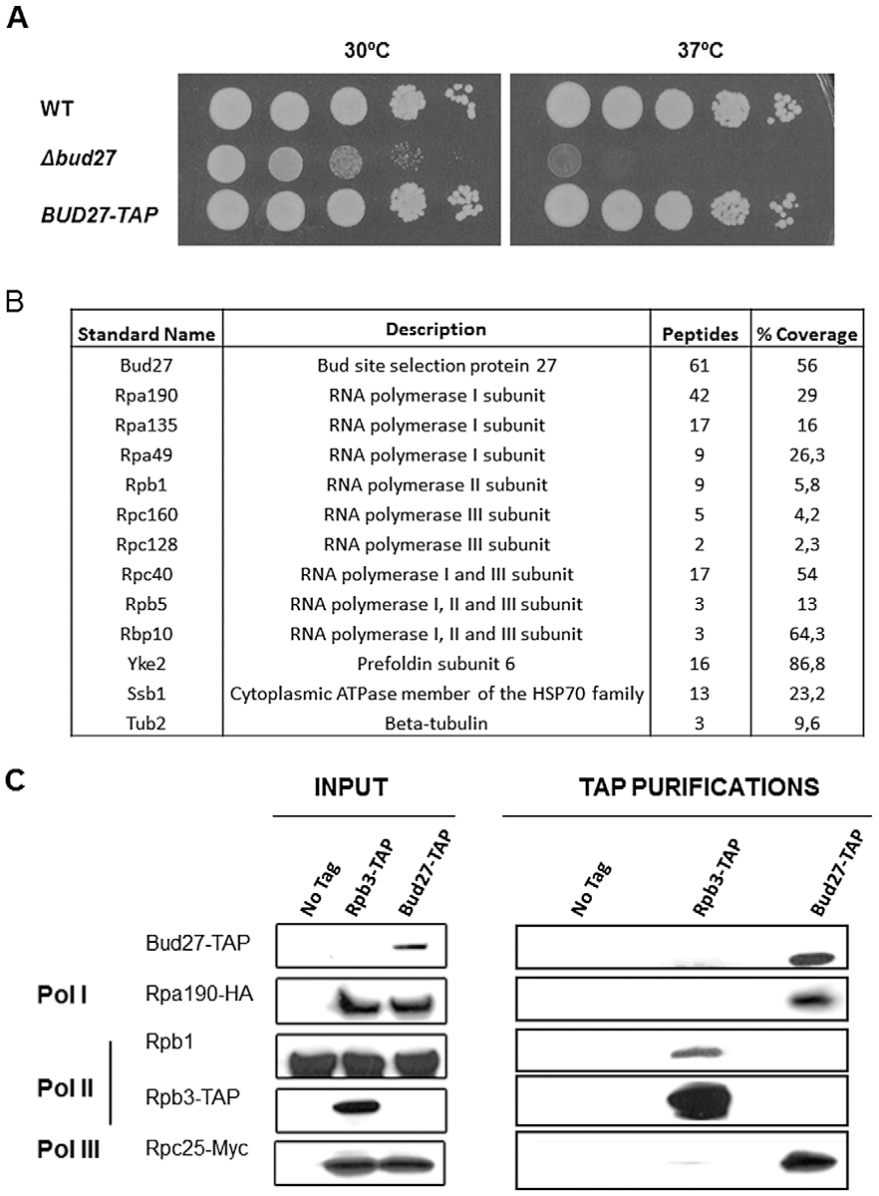
Bud27 physically interacts with the three RNA polymerases. A) Growth of BUD27-TAP, Wild-type and a Δ*bud27* cells inYPD at the indicated temperatures. B) Summary of the proteins interacting with Bud27 in a TAP purification analysis. C) Western blot of protein co-purified with Bud27-TAP (3), Rpb3-TAP (2), and non- tagged wild-type strains (1). anti-HA, anti-Myc, anti-Rpb1 (8WG16), and anti-PAP antibodies were used. No tag: wild-type strain BY4741; Rpb3-TAP: Rpb3-TAP, Rpa190-HA (RNA pol I) and Rpc25-Myc (RNA pol III) strain; Bud27-TAP: Bud27-TAP, Rpa190-HA (RNA pol I) and Rpc25-Myc (RNA pol III) strain.

Immunoprecipitation studies and protein identification by mass spectrometry have shown that URI interacts with components of the RNA pol II in human cells [8], [12]–[14]. In addition, work in yeast [24], [25], using systematic characterization of complexes by TAP and mass spectrometry predicted the association of the URI orthologue, Bud27, with multiple components of the RNA pol II machinery. However, only the physical interaction between Bud27 and Rpb5 in yeast has been further demonstrated and no clear data concerning the association of Bud27 with the other two RNA polymerases have been reported. To gain insights into the association of Bud27 with the three RNA pols and to identify proteins that associate with Bud27 in yeast, we used the *BUD27* gene TAP-tagged at its 3′ end in TAP purifications. As shown, the Bud27-TAP cells grow normally (Figure 1A). Bud27-TAP was affinity-purified from a whole-cell lysate by two consecutive affinity columns (IgG-Sepharose and Calmodulin-Sepharose). After the second purification, 10 proteins were specifically enriched. It bears noting that most of these proteins are members of the three RNA polymerases: Rpa190, Rpa135 and Rpa49 (RNA pol I); Rpc160 and Rpc128 (pol III); Rpc40 (pol I and III); Rpb1 (pol II); Rpb10 and Rpb5 (pol I, II and III) (Figure 1B). In addition, we also identified Yke2 (Pfd6), a member of the Gim/prefoldin protein complex involved in the folding of alfa-tubulin, beta-tubulin, and actin and also a member of the RPAP3/R2TP/prefoldin-like complex participating as intermediary of the RNA pol II assembly in humans [13], [26]. Curiously beta-tubulin and Ssb1, a chaperone member of the HSP70 family [27], were present in the affinity-purified Bud27 preparation.

To confirm the interaction between Bud27 and the three RNA polymerases, we purified Bud27-TAP from a strain containing also Rpa190-HA tagged (RNA pol I) and Rpc25-Myc tagged (RNA pol III) proteins. As shown in Figure 1C, an Rpa190-HA reacting band was revealed. No such band was detected when the TAP purification was performed in control strain BY4741 or in control strain YFN229 containing Rpb3 TAP but also tagged forms of the other two RNA polymerases (Rpa190-HA, and Rpc25-Myc), indicating that Rpa190-HA does not interact with the TAP module and that no anti-Rpa190-HA-reacting material was adsorbed nonspecifically to the beads. Similarly, an Rpc25-Myc reacting band (by using anti-Myc antibodies) was noted when Bud27-TAP was purified, but not in the control strain BY4741 or in a control strain containing Rpb3-TAP. Finally, no reacting band was revealed for Rpb1, although it clearly co-purified in a control strain containing Rpb3-TAP. Thus, we cannot rule out that Bud27 and RNA pol II could associate only transiently *in vivo* or less efficiently. These observations indicate that interactions between Bud27 and the RNA pol I and III are specific, and also suggest that this could similarly account for RNA pol II.

### Bud27 shuttles between the nucleus and cytoplasm in an Xpo1-independent manner

**Figure 2:**
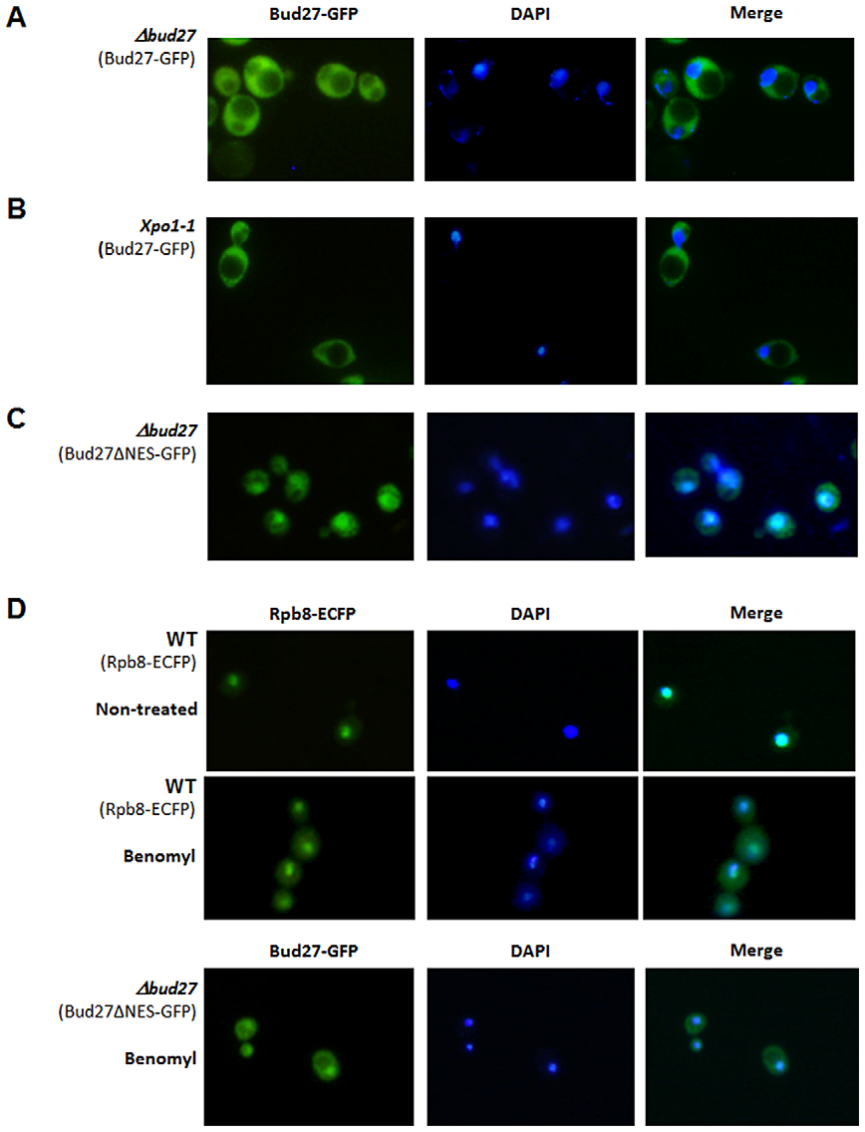
Bud27 shuttles between nucleus and cytoplasm. Live cell imaging of Bud27-Gfp (centromeric plasmid pCM189) in Δ*bud27* mutant cells at 30°C (A) and *Xpo-1* mutant cells for 1 h at 37°C (B). C) Live cell imaging of Bud27ΔNES-GFP in Δ*bud27* mutant cells at 30°C, showing nuclear localization. D) Rpb8-ECFP (C-terminal ECFP tagged Rpb8) and Bud27ΔNES-GFP in wild-type or Δ*bud27* mutant cells treated with benomyl (60 µg/ml) at 30°C.

Our data demonstrating a physical interaction between Bud27 and the three RNA polymerases, as well as the reported localization of Bud27 in the cytoplasm [19], suggest a role for this prefoldin in the cytoplasmic biogenesis of the RNA pols. To clarify the localization of Bud27 in the cell and to explore if this protein shuttles between cytoplasm and nucleus, as is the case in humans and *Drosophila* [20], [21], we used a functional *BUD27-GFP* gene fusion, cloned in a centromeric plasmid, expressed from a Tet-repressible promoter [25]. The functionality of this Bud27-GFP fusion protein was confirmed by its ability to complement the temperature sensitivity of a Δ*bud27* mutant strain (Figure S1). As shown in Figure 2A, Bud27-GFP was preferentially localized at the cytoplasm.

A more detailed analysis of Bud27 amino acid sequence using the NetNES 1.1 and cNLS mapper servers [28], [29] predicted a possible leucine-rich nuclear export signal (NES) between positions 686 and 695 (LRDEIRDFQL) and a nuclear localization signal (NLS; amino acids 562 to 595). Proteins with a NES signal are actively translocated to the cytoplasm via the action of nuclear export pathway mediated by an evolutionarily conserved CRM1/exportin protein Xpo1, suggesting that Bud27 could shuttle between the nucleus and cytoplasm in an Xpo1-dependent manner. To verify this possibility, we examined the localization of Bud27 in *xpo1-1* cells transformed with the above-mentioned plasmid after shifting from 30°C to 37°C for up to 5 h, a condition under which the Xpo1-dependent protein export was blocked [30]. However, no nuclear Bud27-GFP accumulation was found following a shift to 37°C for up to 5 h (Figure 2B).

On the other hand, and as the existence of NES and NLS signals in Bud27 suggest that this is a shuttling protein, we deleted the NES sequence from Bud27-GFP. Our results demonstrated that this led to a nuclear accumulation of Bud27 (Figure 2C). In addition, the deletion of this sequence did not affect the ability of the fusion protein to complement the growth defect caused by the *bud27* null mutation (Figure S1). To gain insight into the mechanism by which Bud27 translocates to the nucleus, we analysed Bud27ΔNES-GFP localization after adding benomyl, a drug demonstrated to promote depolarization of microtubules and accumulation or the largest subunit of the RNA pol II, Rpb1, in the cytoplasm [8]. As expected, benomyl led to the accumulation of RNA pols in the cytoplasm, as shown by monitoring Rpb8-ECFP *in vivo* (Figure 2D upper panel). Contrary, nuclear localization of Bud27ΔNES-GFP is not significantly altered by the addition of benomyl (Figure 2D lower panel).

These data together demonstrate that Bud27 shuttles between nucleus and cytoplasm and suggest that the NES sequence is required for the nuclear export of Bud27 in an Xpo1-independent manner. In addition, the data recorded using benomyl suggest that the translocation of Bud27 to the nucleus is independent of microtubule polarization.

### Lack of Bud27 results in RNA polymerases cytoplasmic accumulation

To help elucidate the function of Bud27 and of its association with the RNA pols, and to investigate the effect of Bud27 in the assembly and/or transport of these enzymes to the nucleus, we tested the hypothesis that Bud27 is needed for correct localization of the three RNA pols. The reason to suspect a role for this prefoldin in the cytoplasmic biogenesis of the three RNA pols came from the observation that Bud27 is localized mainly in the cytoplasm of yeast cells while the three RNA pols in the nucleus.

**Figure 3:**
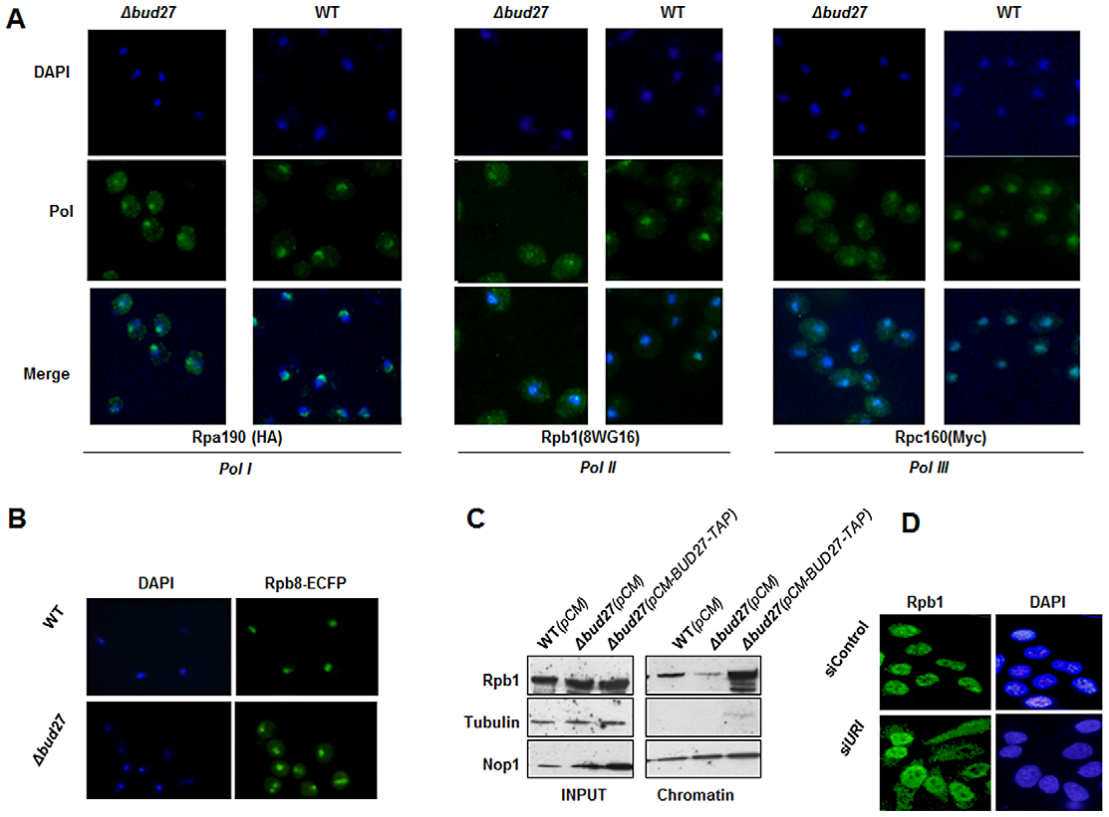
Lack of Bud27 led to RNA pol I, II, and III cytoplasmic accumulation. A) Immunocytochemistry experiments using antibodies against Rpa190-HA (anti-HA), Rpb1 (8WG16), and Rpc160-Myc (anti-Myc) in wild-type and Δ*bud27* mutant cells with tagged Rpa190-HA (RNA pol I) and Rpc160-Myc (RNA pol III), at 30°C. B) Live cell imaging of Rpb8-ECFP in wild-type and Δ*bud27* mutant cells, at 30°C. C) Western blot of chromatin fractions from wild-type and Δ*bud27* mutant cells harbouring an empty vector (pCM) or a vector overexpressing *BUD27* (*pCM-BUD27-TAP*). Tubulin and Nop1 were used as controls of non-chromatin and chromatin fractions, respectively. D) Rpb1 immunolocalisation analysis (8WG16) in human pulmonary fibroblast under silencing of URI. As a control, cells without siRNA heteroduplex.

Then we performed immunocytochemistry experiments in a wild-type and a Δ*bud27* mutant strain containing an Rpa190-HA tagged (RNA pol I) and Rpc160-Myc tagged (RNA pol III) proteins, using anti-HA, anti-Rpb1 (8WG16) and anti-Myc antibodies, to analyse the intracellular localization of the largest subunits of the RNA pol I, II, and III, respectively. In a wild-type strain (Figure 3A), fluorescence for Rpb1 and Rpc160 was restricted to the nucleus, while for Rpa190 it was mainly nucleolar, indicating that, as expected, RNA pol II and III were localized in the nucleus and RNA pol I, mainly in the nucleolus. However, deletion of *BUD27* resulted in the accumulation of the three RNA pols in the cytoplasm, although nuclear and nucleolar, but more diffuse, staining was also observed. To corroborate these results and to monitor the localization of another subunit shared by the three RNA polymerases, we genomically tagged Rpb8 with a C-terminal ECFP tag and we monitored its localization by live cell imaging (Figure 3B). As in the case for Rpa190, Rpb1, and Rpc160, fluorescence for Rpb8 was detected mainly in the nucleus of a wild-type strain, while a clear cytoplasmic accumulation was found in a Δ*bud27* mutant strain. These results indicate that the three RNA enzymes are partially mislocalized, according to the differences in the amount of Rpb1 associated with chromatin fractions (Figure 3C). In addition, nuclear localization of the three RNA pols seems not to depend on Bud27 localization, since Bud27 variant that lacks NES sequence and that is accumulated in the nucleus did not impair the nuclear localization of the RNA pol I, II or III (Figure S2A).

Also, we investigated whether cells resumed growth and RNA pols nuclear localization when a Δ*bud27* mutant strain was complemented with a *BUD27-TAP* gene fusion into a centromeric plasmid expressed from a Tet-repressible promoter [25]. As expected, *BUD27-TAP* fully restored growth of the Δ*bud27* mutant strain at the restrictive temperature of 37°C (Figure S1). In addition, Rpb8-ECFP signal was again restricted to the nucleus, suggesting that the three RNA pols again became nuclear (Figure S2B). Furthermore, these data correlate with an increase in the amount of Rpb1 associated with chromatin fractions in the Δ*bud27* mutant strain overexpressing the *BUD27-TAP* gene fusion (Figure 3C).

All together, these data indicate that Bud27 is necessary for correct nuclear localization of RNA pols in the *S. cerevisiae* nucleus, and suggest that it may play a role in assembly and/or nuclear transport of the three enzymes.

### Silencing of URI results in cytoplasmic accumulation of RPB1 in human cells

URI is an evolutionarily conserved member of the prefoldin family among eukaryotes [18], [22]. Then, to start elucidating if in humans URI IS also involved in the biogenesis of the RNA pols, we performed siRNA silencing experiments in human pulmonary fibroblast and monitored the effect of URI depletion on Rpb1 intracellular localization. As shown by q-RT PCR, URI mRNA expression decreased to 40% at 100 nM of siURI (Figure S3). Furthermore, silencing of URI resulted in the accumulation of Rpb1 in the cytoplasm of treated cells (Figure 3D), as revealed by immunocytochemistry experiments using 8WG16 antibodies. Contrary, control experiment did not affect nuclear localization of Rpb1. These data suggest that, as it is the case for Bud27, URI modulates the translocation of RNA pol II to the nucleus, pointing to a common role for these conserved proteins in the biogenesis of, at least, the RNA pol II.

### Bud27 is required for correct assembly of the three RNA polymerases

**Figure 4:**
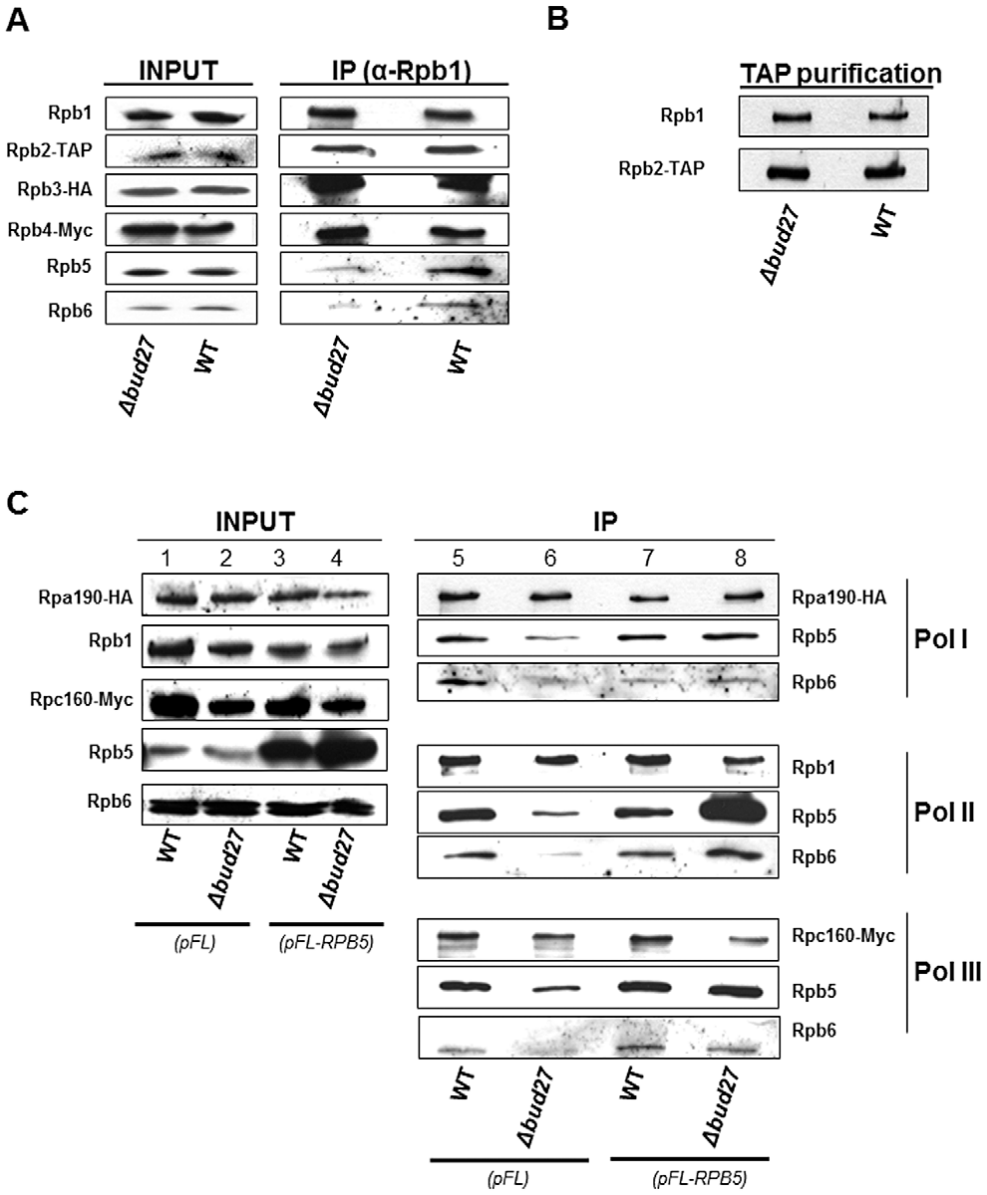
Lack of Bud27 affects assembly of RNA pol I, II, and III. A) RNA pol II was immunoprecipitated with anti-Rpb1 antibodies (8WG16) from a strain containing Rpb2-TAP, Rpb3-HA and Rpb4-Myc. RNA pol II subunits were analysed with Anti-HA, anti-Rpb1 (8WG16), anti-Myc, anti-PAP, anti-Rpb5 and anti-Rpb6 antibodies. B) Western blot of Rpb1 co-purified with RPB2-TAP. Anti-Rpb1 (8WG16) and anti-PAP antibodies were used. C) RNA pol I, II and III were immunoprecipitated with anti-HA, anti-Rpb1 (8WG16) and anti-Myc antibodies from a wild-type and a Δ*bud27* mutant strains containing Rpa190-HA (RNA pol I) and Rpc160-Myc (RNA pol III), transformed with a plasmid overexpressing RPB5 (*pFL-RPB5*), or with an empty vector (*pFL*). Rpa190-HA, Rpb1, Rpc160-Myc, Rpb5, and Rpb6 were analysed by Western blot with the antibodies indicated above.

To investigate the effect of Bud27 in RNA pols complex assembly, we immunoprecipitated RNA pol II from a wild-type and a Δ*bud27* mutant strain containing functional tagged versions of different RNA pol II subunits (Rpb2-TAP, Rpb3-HA and Rpb4-Myc). We performed immunoprecipitation experiments using anti-Rpb1 antibodies (8WG16) and analysed different RNA pol II subunits corresponding to the different assembly intermediate previously described, Rpb1 together with Rpb4/5/7/8/9 and Rpb2 together with Rpb3/10/11/12 [10], [12], [31]. Our results revealed no significant differences in the yield or subunit composition between the two polymerases for Rpb1, Rpb2, Rpb3, or Rpb4 (Figure 4A). It is worth noting that Rpb2 purification gave also similar amount of Rpb1/Rpb2 (Figure 4B).

Also, we analysed Rpb5, a common subunit shared by the three RNA polymerases and demonstrated by us and others [18] to physically interact with Bud27. Surprisingly, the amount of Rpb5 clearly decreased in RNA pol II immunoprecipitated from a Δ*bud27* mutant strain (Figure 4A), indicating that lack of Bud27 affects RNA pol II assembly. To corroborate these results and to investigate whether Bud27 also participates in the assembly of the other two RNA pols (I and III), we immunoprecipitated the three RNA pols from a wild-type and a Δ*bud27* mutant strain containing functional tagged versions of different RNA pols subunits (Rpa190-HA and Rpc160-Myc). The immunoprecipitation of the three largest RNA pol subunits, using anti-HA, anti-Rpb1 (8WG16), and anti-Myc antibodies, again revealed significant differences in yield between Rpb5 and the largest subunits of the three RNA pols between wild-type and mutant polymerase complexes (Figure 4C, lines 5 and 6 ).

Rpb6, another common subunit to the three RNA pols seems to assemble rather late in humans although this mechanism is unclear [12]. Thus, we also analysed the amount of Rpb6 in immunoprecipitated RNA pol II between the wild-type and Δ*bud27* mutant strains. Again, surprisingly, the amount of Rpb6 clearly decreased in RNA pol II mutant complex (Figure 4A and 4C, lines 5 and 6), but also in RNA pol I and III (Figure 4C, lines 5 and 6). These data indicate that Bud27 has a role in the assembly of the three RNA pols, by interfering with the correct assembly of Rpb5 and Rpb6 in the complexes. Furthermore, the fact that no significant differences were detected between the wild-type and the mutant strain in whole-cell extracts for any of the RNA pols subunits analysed suggests that when Bud27 lacks, the non-assembled subunits are not rapidly degraded.

To extend our analysis, we performed Rpb3-TAP purification from wild type and Δ*bud27* mutant containing functional tagged version of Rpb3 (Rpb3-TAP), as Rpb3-TAP purification has been largely and successfully used to purify RNA pol II [32]–[34]. The protein mixture obtained in each case was subjected to multidimensional protein identification technology (MudPIT) [35] and to separation by gel electrophoresis. From our analysis we can conclude that all RNAPII subunits are associated to Rpb3 in absence of Bud27 (Table S1), but however, the yield of Rpb3 recovery drops abruptly in Δ*bud27* cells. It is also significant the reduction of Rpb3 co-purifying proteins. Taking together, our results suggest that lack of Bud27 led to an instable RNA pol II complex and then, that Bud27 is also necessary to maintain stability of the enzyme.

### *RPB5* overexpression corrects temperature sensitivity, rapamycin sensitivity, nuclear RNA pols localization, and RNA pols assembly in cells lacking Bud27

The data above strongly indicate that Bud27 is required for assembly of the three RNA pols and that this mechanism depends on correct assembly or stabilization of Rpb5. Because Rpb5 was identified as an interactor with Bud27, we considered the possibility that these proteins are functionally linked.

**Figure 5:**
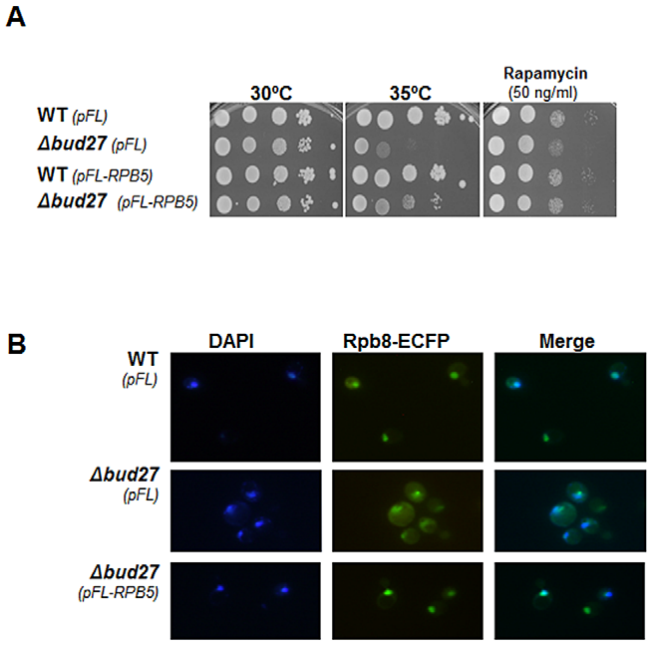
Δ*bud27 mutant* phenotypes are corrected by overexpression of different *BUD27* constructions and *RPB5*. A) Growth of wild-type and Δ*bud27* mutant strains transformed with vectors overexpressing *RPB5*, at different temperatures or in the presence of rapamycin. *pCM* and *pFL* correspond to the control empty vectors. B) Live cell imaging of Rpb8-ECFP in wild-type and Δ*bud27* mutant cells at 30°C, containing empty vector (*pFL*) or overexpressing *RPB5* (*pFL-RPB5*).

In an attempt to clarify the relationship between Rpb5 and Bud27, we explored whether *RPB5* overexpression corrects the temperature sensitivity of the Δ*bud27* mutant strain. Notably, increasing dosage of Rpb5 suffices to rescue the growth defect of the strain lacking Bud27 at a restrictive temperature (Figure 5A), as is the case for *BUD27* overexpression (Figure S1). Bud27 in yeast and URI in human cells were originally characterized as regulators of gene expression controlled by TOR (for target of Rapamycin) pathway [18]. As opposed to a *S. cerevisiae* wild-type strain, the Δ*bud27* mutant was slightly sensitive to Rapamycin (Figure 5A). Again, *RPB5* overexpression corrected the sensitivity of the Δ*bud27* mutant cells to this drug (Figure 5A).

Considering the functional connection between *BUD27* and *RPB5*, we investigated whether increasing Rpb5 dosage could also correct Bud27-dependent RNA pols localisation. Then we monitored the localization of the three RNA pols by analysing Rpb8-ECFP by live cell imaging. Rpb8 was observed mainly in the nucleus when *RPB5* was overexpressed, as compared to the same strain containing a void plasmid as a control (Figure 5B), as in the case of *BUD27* overexpression (Figure S1). Similar results were found by performing immunolocalization experiments using anti-HA, anti-Rpb1 (8WG16) and anti-Myc antibodies in a Δ*bud27* mutant strain containing functional tagged versions of different RNA pols subunits (Rpa190-HA and Rpc160-Myc; Figure S4). Taken together, our data showing that lack of Bud27 does not impair the nuclear localization of RNA pols under *RPB5* overexpression, suggest that Bud27 is not the main requisite for their nuclear import.

Also, we investigated whether *RPB5* overexpression was sufficient to rescue the defects in RNA pols assembly observed in the Δ*bud27* mutant strain that could also explain the correction in RNA pols mislocalization. We immunoprecipitated the three RNA pols from a wild-type and a Δ*bud27* mutant strain containing functional tagged versions of different RNA pols subunits (Rpa190-HA and Rpc160-Myc) under *RPB5* overexpression. Immunoprecipitation of the three largest RNA pols subunits, using anti-HA, anti-Rpb1 (8WG16) and anti-Myc antibodies, revealed no significant differences in the yield between Rpb5 and the largest subunits of the three RNA pols between wild-type and mutant polymerase complexes (Figure 5B) with respect to the same strains harbouring a void plasmid as a control. In addition, similar results were found for Rpb6. These results demonstrate that increasing dosage of Rpb5 is sufficient to correct the RNA pols assembly defects caused by the lack of Bud27, suggesting that it accounts for the rescue of nuclear RNA pols localization.

### Bud27 Rpb5-binding domain is required for nuclear RNA pols localization, although both Prefoldin and Rpb5-binding domains are dispensable for growth

To test which conserved domains of Bud27 are important for growth, we transformed Δ*bud27* cells with plasmids expressing full-length Bud27 or proteins deleted for the indicated domains, and tested their ability to complement the temperature-sensitive phenotype (Figure S1). Surprisingly, neither the PFD or the Rpb5-binding domain nor both were functionally important *in vivo*, as previously indicated [19].

**Figure 6:**
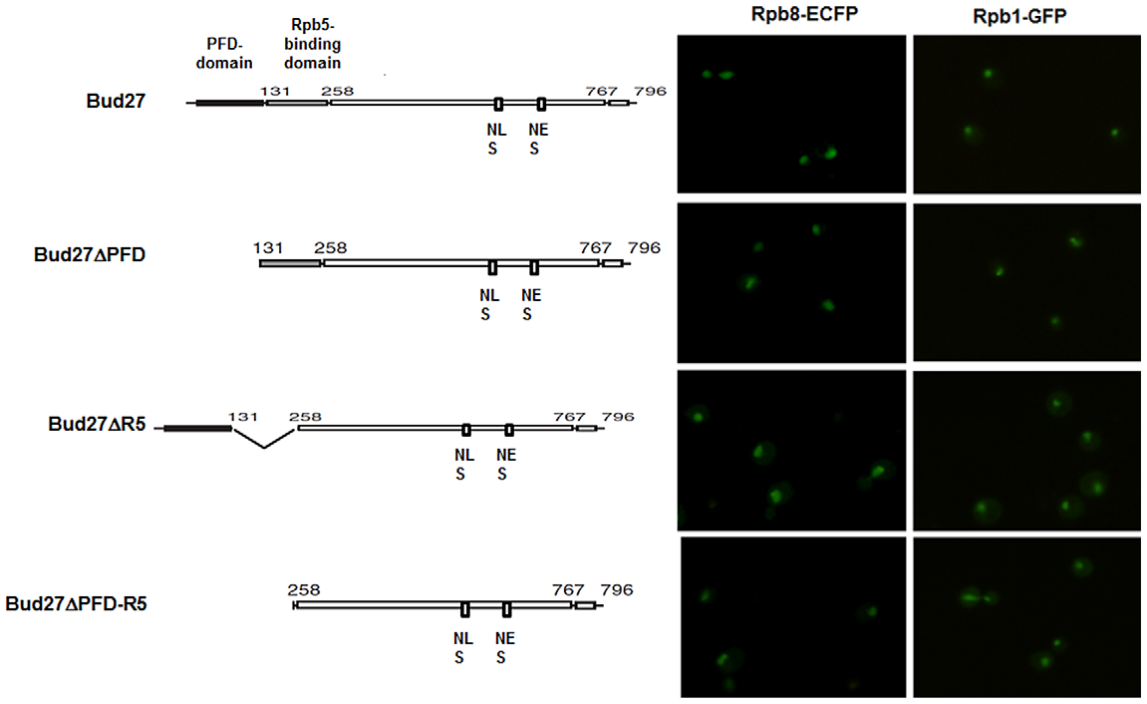
PFD, Rpb5-binding domains, or both are dispensable for RNA pol I, II, and III nuclear localization. Live cell imaging of Rpb8-ECFP and Rpb1-GFP in Δ*bud27* mutant cells at 30°C, containing vectors overexpressing whole *BUD27* or *BUD27* deleted for PFD, Rpb5-binding domains or both.

To assess whether the above-mentioned conserved domains participated in the nuclear localization of the three RNA pols, we monitored Rpb8-ECFP and Rpb1-GFP by live cell imaging in a Δ*bud27*cells with plasmids containing full-length *BUD27* or *BUD27* deleted for the PFD, Rpb5-binding domains, or both. As shown in Figure 6, deletion of Rpb5-binding domain led to a clear Rpb1-GFP cytoplasmic accumulation. Similarly, a more diffuse cytoplasmic Rpb8-ECFP signal was observed. However, PFD-binding domain was not crucial for the correct nuclear localization of the three RNA pols. Thus, we conclude that nuclear localization of the three RNA pols mediated by Bud27 is dependent on Rpb5-binding domain.

### The role of Bud27 in RNA pols biogenesis is independent of the chaperone prefoldin (PFD) complex

URI (Bud27) is believed to function as a scaffold protein able to assemble additional members of chaperone prefoldin (PFD) family through its PFD and Rpb5-binding domains in both human and yeast. Human and yeast PFD is a complex composed of six different subunits, PFD1-PFD6, referred to as the prefoldin/GimC complex, which functions as a molecular chaperone and delivers newly synthesised unfolded proteins to cytosolic chaperonin TRiC/CCT to facilitate the folding of proteins [13], [23]. In humans, PFDs and URI are constituents of an 11-subunit complex associated to the RNA pol II, namely the RPAP3/R2TP/prefoldin-like complex, suggested to participate in RNA pol II assembly [12], [13]. In addition, data from yeast and mammalian cells have shown that the functions of the prefoldin and CCT chaperone complexes are eliminated by removal of individual subunits [36].

**Figure 7:**
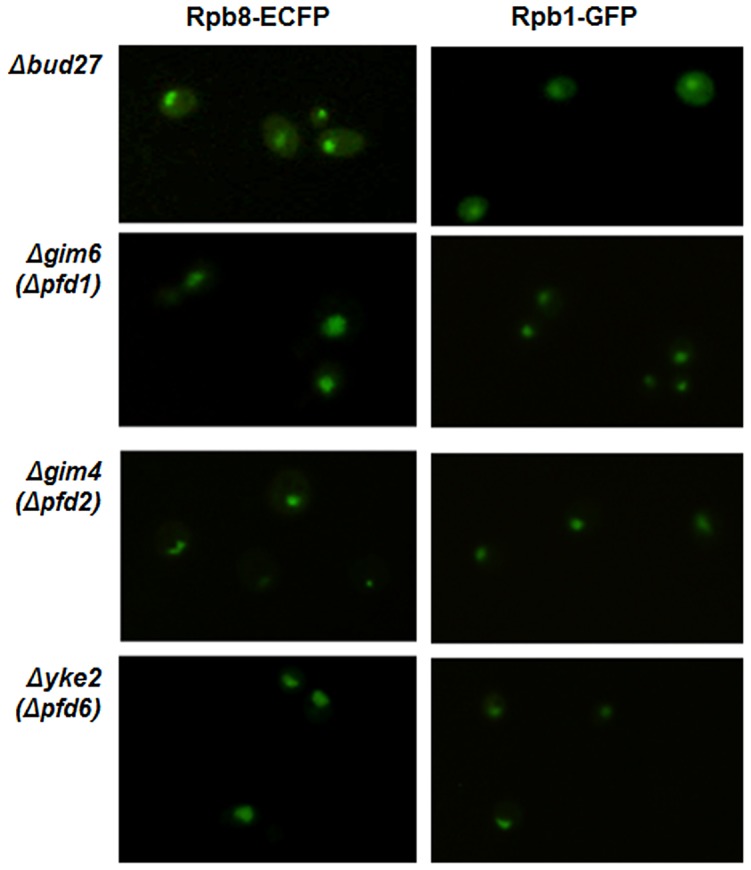
RNA pol I, II, and III nuclear localization is independent of the chaperone prefoldin (PFD) complex. Live cell imaging of Rpb8-ECFP and Rpb1-GFP in Δ*bud27*, Δ*gim6* (Δ*pfd1*), Δ*gim4* (Δ*pfd2*), and Δ*yke2* (Δ*pfd6*) mutant cells containing C-terminal ECFP tagged Rpb8 and C-terminal GFP tagged Rpb1, at 30°C.

To evaluate whether the deletion of other components of the prefoldin complex in yeast also participate in the RNA pols nuclear localization, we analysed Rpb8-ECFP and Rpb1-GFP localization by live cell imaging in strains deleted for different prefoldins: *YKE2* (*PFD6*), shown by us and others [23] to physically interact with Bud27, with Rpb5 [24] and genetically with other components of the RNA pol I and III machinery [37], [38]; *GIM6* (*PFD1*), which genetically interacts with *BUD27* [19] and with subunits of RNA pol I and III machinery [37], [39], [40]; GIM4 (PFD2), which interacts neither with *BUD27* nor with the RNA pols. Surprisingly, in contrast to Bud27, these three components of the prefoldin complex are dispensable for the nuclear localization of the three RNA pols at 30°C or even at 37°C (Figure 7, for 30°C), indicating that the role of Bud27 in the biogenesis of the RNA pols seems to be independent from the rest of the prefoldin complex.

### The role of Bud27 in RNA pols biogenesis is independent of Iwr1

**Figure 8:**
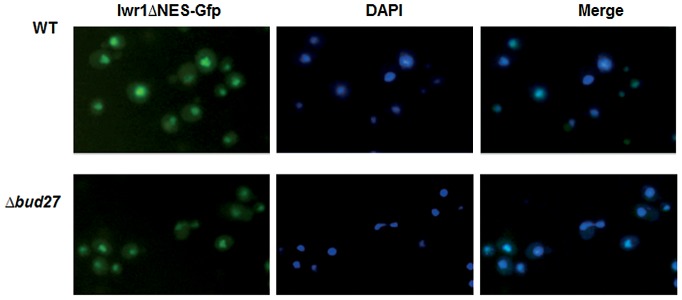
Iwr1 nuclear localization is independent of Bud27. Live cell imaging of Iwr1-Gfp deleted for its NES domain in wild-type and Δ*bud27* cells at 30°C.

The conserved protein Iwr1 was originally identified as a protein that co-purified with almost every subunit of RNA pol II and that interacts with the basal transcription machinery and regulates the transcription of specific genes [30]. Recently, it has been shown that Iwr1 specifically binds RNA pol II between Rpb1 and Rpb2 and directs its nuclear import, although it is not involved in RNA pol I or III transport [6], [10]. Curiously, *IWR1* genetically interacts with *BUD27* [41] and *RPB5* [42] and physically with Bud27 [24] although we could not reproduce this interaction by our TAP purification. Based on these data, and to rule out the possibility that accumulation of RNA pol II in the cytoplasm in cells lacking Bud27 is not an indirect effect due to the mislocation of Iwr1, we analysed localization of Iwr1 in *Δb*ud27 cells. For this experiment, cells were transformed with a plasmid expressing an Iwr1 protein lacking its NES domain and shown to accumulate in the nucleus, instead of a plasmid with entire *IWR1*, since it leads to a diffuse signal and no clear nuclear localization [30]. As shown (Figure 8), deletion of *BUD27* does not affect the nuclear localization of Iwr1-ΔNES. These data suggest that nuclear localization of Iwr1 does not require interaction with Bud27.

## Discussion

Human URI/RMP and its orthologue in yeast, Bud27, is the most studied member of the prefoldin-like (PFD) family of ATP-independent molecular chaperones, also called unconventional prefoldin Rpb5 interactor [18]. Based on recent data of large-scale proteomic screen of RNA polymerases in human cells, URI has been shown to be a component of the HSP90/R2TP complex and has been proposed to participate in the biogenesis of RNA polymerases [8], [12]. However, this role has not been confirmed. In this work, we show evidence that Bud27 in *S. cerevisiae* is the first example of a protein that participates in the biogenesis of all RNA polymerases, and mediates the assembly of the three transcriptional complexes. Furthermore, Bud27 seems not to play a role in the nuclear transport of the RNA pols. Our data also suggest similar role for human URI.

Consistent with a role for Bud27 in RNA pols biogenesis, we demonstrated by TAP purification and immunoprecipitation experiments that Bud27 interacts with different subunits of the three RNA pols. Our data agrees with those of Krogan et al. showing physical interactions between Bud27 and RNA pol II [24] or between URI or Bud27 and Rpb5, a common subunit shared by the three RNA pols [14], [18]. Moreover other authors identified, in human cells, URI as a component of the R2TP/prefoldin-like complex which binds the largest subunits of the RNA pol II [12], [43]. Finally, in accordance with these physical associations, genetic interactions between *BUD27* and different components of the three transcriptional machineries have been found in yeast by us (Mirón-García, unpublished data) and others [44].

Biochemical and structural studies of RNA pols have proposed a detailed model of these enzymes, but however, little is known on how they assemble into the complexes or how they are transported from the cytoplasm to the nucleus. Our work provides new data to elucidate the mechanisms governing RNA pols biogenesis and localization and complement those in human and yeast [6]–[8], [12]. Bud27 is the first protein so far demonstrated to participate in the biogenesis of the three RNA pols, modulating their assembly. Accumulation of the three RNA pols in the cytoplasm, as a consequence of *BUD27* deletion accounts for a defect in nuclear transport. However, lack of Bud27 does not impair RNA pols nuclear localization under *RPB5* overexpression, suggesting that Bud27 is dispensable for their nuclear import. Only two proteins in *S. cerevisiae* had been shown to participate in RNA pol II biogenesis so far, Iwr1 and Npa3, and only the Npa3 homologue RPAP4/GPN1 in humans. Notably, these proteins are necessary for nuclear transport but none of them are involved in pol assembly [6]–[8].

Our immunoprecipitation experiments in Δ*bud27* mutant strain and the analysis of different subunits corresponding to the different assembly intermediate previously described [12], [31], demonstrate that Bud27 mediates the assembly of the three RNA pols. In fact, the lack of Bud27 alters the correct assembly of Rpb5 and Rpb6 in the three RNA complexes and led to a more instable enzyme. Results are also consistent with RNA pols assembly in the cytoplasm as a prerequisite for their nuclear import and agree with recent observations in yeast and humans [6], [8], [12]. In addition, co-purification and gel filtration analysis (unpublished data) suggest that RNA pols intermediaries do not appear in yeast in the absence of Bud27. These data, together with the fact that only differences in the yield of Rpb5 and Rpb6 were observed and that *RPB5* overexpression corrects not only assembly but also nuclear RNA pols transport, point to the fact that Bud27 mediates RNA pols assembly in an Rpb5-dependent manner, in agreement with data confirming its role in protein folding [23]. Our data point to a role of Bud27 in the correct folding of Rpb5 to the rest of the complex, since lack of Bud27 leads to transcription defects not related to a decrease in nuclear RNA pols amount but to RPB5-dependent processes (Mirón-García, in preparation). Furthermore, we cannot rule out that Bud27 could act to stabilize the interaction between Rpb5 and the rest of the RNA pols complexes, and hence their integrity. Interestingly, the Rpb5-binding domain but not the conserved PFD-binding domain of Bud27 is essential for this role, since only Δ*bud27* cells expressing a version of Bud27 lacking the Rpb5-binding domain show cytoplasmic RNA pols localization. In addition, the defect in Rpb6 assembly is also consistent with the fact that *RPB6* overexpression partially corrects the temperature sensitivity of the Δ*bud27* mutant (our unpublished data) and with the physical contact between Rpb5 and Rpb6 on the RNA pol II structure [45]. Furthermore, as *BUD27* is not essential and its deletion seems only to affect part of the RNA pols complexes, it appears that Bud27 participates in coordination with other proteins to address its role in RNA pols biogenesis. These results also provide information concerning the mechanisms governing the assembly of Rpb5 and Rpb6 into the rest of the complexes. Our data suggest that Rpb6 could assemble rather late once Rpb5 is assembled, as previously proposed in human [12]. However, we cannot disregard the possibility that it assembles before and Rpb5 would be necessary to stabilize its contact with the other components of the RNA pols. Alternatively, we cannot rule out that Rpb5 could be assembled in early steps of the RNA pols biogenesis participating in maintaining the integrity of the RNA pols, although this possibility seems less unlikely.

As is the case for Bud27 in yeast, HSP90 and its cochaperone RPAP3 in human cells have been shown to coordinate the assembly of RNA pol II and its involvement in RNA pol I and III assembly has been suggested [43]. Curiously, HSP90 and RPAP3 are members of the RTP2/prefoldin-like complex, a molecular machine dedicated to the assembly of multi-molecular protein complexes, such as the RNA pol II. It contains 11 components, among them URI and Rpb5. On the contrary, in yeast the R2TP complex contains five proteins and Rpb5 and Bud27 have not been found as bona-fide constituents [43]. Interestingly, prefoldin 6 (Yke2), which is part of the human RTP2 complex also appears as a Bud27 interactor when purified via TAP. Thus is temptating to speculate that in yeast, also Bud27 and Rpb5 could associate with the RT2P complex. Moreover, physical interactions between Bud27 and a member of the Hsp70 family chaperones (Ssb1), as well as with the beta-tubulin chain of the microtubules (Tub2) have been identified (Figure 1B). This is consistent with previous data from two-hybrid analysis [19], [23]. Notably, polymerization of tubulins into microtubules requires prefoldins and chaperonin CCT complex, which has been shown to interact with RNA pol II subunits [8], [36]. Impairing microtubule assembly, both in humans and yeast, leads to a RNA pol II mislocalization in the cytoplasm [8]. Thus it is possible that these interactions are functional, althought further work will be necessary to address this issue.

As shown here RNA pols nuclear localization is dependent on Rpb5-binding domain. However, Bud27 PFD and Rpb5-binding conserved domains are not required for growth, neither for its role in translation [19]. Then, these domains could be also important for other roles of Bud27 in the nucleus, such as for its interaction with other transcriptional regulators [14], [16], [20]. Consistent with this possibility Bud27 shuttles between the cytoplasm and nucleus via an Xpo1-independent pathway. Discrepant results have been reported concerning Bud27 localization. According to Desplaces et al., Bud27 is excluded from the nucleus in yeast [19]. However, in humans and *Drosophila* [20], [21] nuclear localization for Bud27 has also been reported. Moreover, physical association between Rpb5, Bud27, and transcription factors TFIIF, as well as between Rpb5 and TFIIB [14], [16], let us to propose that Bud27 could compete with these transcription factors to bind Rpb5.

Finally, silencing experiments in human cells, account for a conserved role of URI in RNA pols biogenesis, suggesting similar mechanisms that must be deciphered.

## Materials and Methods

### Yeast strains, plasmids, genetic manipulations, media, and genetic analysis

Common yeast media, growth conditions, and genetic techniques were used as described elsewhere [46]. Rapamycin (LCLAbs, USA) and Benomyl (Sigma-Aldrich) was used at the indicated concentrations.

Strains, plasmids and primers are listed in Table 1, Table 2, and Table 3.

**Table 1:** *S. cerevisiae* strains.

Strain	Genotype	Origin
BY4741	*MATa his3Δ1 leu2Δ0 met15Δ0 ura3Δ0*	Euroscarf
BY4742	*MATα his3Δ1 leu2Δ0 lys2Δ0 ura3Δ0*	Euroscarf
Y01246	*MATα his3Δ1 leu2Δ0 lys2Δ0 ura3Δ0 YJL179W(GIM6, PFD1)::kanMX4*	Euroscarf
Y00243	*MATα his3Δ1 leu2Δ0 lys2Δ0 ura3Δ0 YEL003W(GIM4,PFD2)::kanMX4*	Euroscarf
Y04149	*MATa his3Δ1 leu2Δ0 met15Δ0 ura3Δ0 YLR200W(YKE2, PFD6)::kanMX4*	Euroscarf
Y05642	*MATa his3Δ1 leu2Δ0 met15Δ0 ura3Δ0 YFL023W(BUD27)::kanMX4*	Euroscarf
ATCC201388 (*BUD27-GFP*)	*MATa his3Δ1 leu2Δ0 met15Δ0 ura3Δ0 YFL023W(BUD27)::GFP::HIS3MX6*	Invitrogen
YSC1178 (*BUD27-TAP*)	*MATa his3Δ1 leu2Δ0 met15Δ0 ura3Δ0 YFL023W(BUD27)::TAP::HIS3MX6*	Open Biosystems
YSC1178 (*RPB2-TAP*)	*MATa his3Δ1 leu2Δ0 met15Δ0 ura3Δ0 YOR151C(RPB2)::TAP::HIS3Mx6*	Open Biosystems
W303-1A	*MATa ade2-1 his3-11,15 leu2-3,112 trp1-1 ura3-1 can1-100*	[52]
W303-1B	*MATα ade2-1 his3-11,15 leu2-3,112 trp1-1 ura3-1 can1-100*	[52]
YPH500	*MATα ade2-101 his3-Δ200 leu2Δ1 lys2-801 trp1-Δ63 ura3-52*	[53]
MW3522	*MATa ade2-101 his3-Δ200 leu2-Δ1 lys2-801 trp1-Δ63 ura3-52 YOR341W(RPA190)::3HA::HIS3*	[49]
XPO1	*MATa ade2-1 can1-100 his3-11,15 leu2-3,112 trp1-1 ura3-1 YGR218W(XPO1)::LEU2 [pKW440 (XPO1 in pRS313)]*	[54]
xpo1-1	*MATa ade2-1 can1-100 his3-11,15 leu2-3,112 trp1-1 ura3-1 YGR218W(XPO1)::LEU [pKW457 (xpo1-1 in pRS313)]*	[54]
YVV50-4c	*MATa ade2-101 his3-Δ200 leu2Δ1 lys2-801 trp1-Δ63 ura3-52 YIL021W(RPB3)::HA::KanMX4*	[55]
D473-4A	*MATα ade2-1 his3-11,15 leu2Δ1 lys2-801 trp1-Δ63 ura3-1 YBR154C(RPB5)::URA3::LEU2 [ pFL44L-RPB5]*	Gift from P. Thuriaux
LMY3.1	*MATa his3Δ1 leu2Δ0 met15Δ0 ura3Δ0 YDR007W(TRP1)::kanMX4; YJL140W(RPB4)::Myc18::TRP1*	Gift from S. Chávez
MW3608	*MATα ade2-101 his3-Δ200 leu2-Δ1 lys2-801 trp1-Δ63 ura3-52 YOR116C(RPC160)::13Myc::TRP*	Gift from M. Werner
SL876a	*MATa his3-Δ200 leu2-3,112 trp1-Δ63 ura3-52 YIL021W(RPB3)::TAP::KIURA*	Gift from P. Thuriaux
YCZ106	*MATα ade2-101 his3-Δ200 leu2Δ1 lys2-801 trp1-Δ63 ura3-52 YKL144C(RPC25)::Myc::KanMX6*	Gift from P. Thuriaux
D495-1c	*MATa his3-Δ200 leu2-Δ1 lys2-801 trp1-Δ63 ura3-52 YKL144C(RPC25)::Myc::KanMX6 YIL021W(RPB3)::TAP::URA3 YOR341W(RPA190)::3HA::HIS3*	This workYFN56×YCZ106
D610-11A	*MATα his3Δ1 leu2-3,112 lys2-801 met15Δ0 trp1-Δ63 ura3-1 YFL023W(BUD27)::kanMX4 YBR154C(RPB5)::URA3::LEU2 [ pFL44L-RPB5]*	This workY05642×D473-4A
YFN56	*MATa his3-Δ200 leu2-3,112 lys2-801 trp1-Δ63 ura3-52 YIL021W(RPB3)::TAP::KIURA YOR341W(RPA190)::3HA::HIS3*	This workSL876a×MW3522
YFN105	*MATa his3Δ1 leu2Δ0 met15Δ0 ura3Δ0 trp1-Δ63 YFL023W(BUD27)::kanMX4*	This workBY4741×D610-11A
YFN106	*MATα his3Δ1 leu2-3,112 lys801 met15Δ0 ura3Δ0 trp1-Δ63 YFL023W(BUD27)kanMX4*	This workBY4741×D610-11A
YFN223	*MATα ade2-101 his3-Δ200 leu2-Δ1 lys2-801 trp1-Δ63 ura3-52 YOR341W(RPA190)::3HA::HIS3 YOR116C(RPC160)-13Myc::TRP*	This workD495-1c×MW3608
YFN229	*MATα ade2-1 his3-11,15 leu2-3,112 lys2-801 trp1-1 ura3-1 YKL144C(RPC25)::Myc::KanMX6 YOR341W(RPA190)::3HA::HIS3 YIL021W(RPB3)::TAP::URA3*	This workD495-1c×W303-1A
YFN233	*MATa ade2-101 his3-Δ200 leu2Δ0 trp1-Δ63 ura3Δ0 YOR341W(RPA190)::3HA::HIS3 YOR116W(RPC160)-13Myc::TRP YFL023W(BUD27)::kanMX4*	This workYFN223×YFN105
YFN255	*MATa ade2-1 his3-11,15 leu2Δ0 lys2-801 trp1-1 ura3-1 YKL144C(RPC25)::Myc::KanMX6 YOR341W(RPA190)::3HA::HIS3 YFL023W(BUD27)::TAP::HIS3MX6*	This workYFN229×YSC1178 (*BUD27-TAP*)
YFN289	*MATα his3-11,15 leu2-3,112 met15Δ0 trp1-1 ura3-1 YOR151C(RPB2)::TAP::HIS3Mx6*	This workYSC1178(*RPB2-TAP*)×W303-1B
YFN308	*MATa his3Δ1 leu2Δ0 met15D0 trp1-1 ura3Δ0 YOR151C(RPB2)::TAP::HIS3Mx6 YJL140W(RPB4)::Myc18::TRP1*	This workYFN289×LMY3.1
YFN323	*MATα ade2-101 his3Δ1 leu2-3,112 lys2-801 ura3-52 YFL023W(BUD27)::kanMX4 YIL021W(RPB3)::HA::KanMX4*	This workYFN106×YVV50-4c
YFN329	*MATα his3Δ1 leu2-3,112 lys2-801 trp1-Δ63 ura3-52 YFL023W(BUD27)::kanMX4 YIL021W(RPB3)::HA::KanMX4 YOR151C(RPB2)::TAP::HIS3Mx6 YJL140W(RPB4)::Myc18-TRP1*	This workYFN323×YFN308
YFN331	*MATa his3Δ1 leu2-3,112 lys2-801 trp1-Δ63 ura3Δ0 YIL021W(RPB3)::HA::KanMX4 YOR151C(RPB2)::TAP::HIS3Mx6 YJL140W(RPB4)::Myc18-TRP1*	This workYFN323×YFN308
YFN334	*MATa his3Δ1 leu2Δ0 met15Δ0 trp1-Δ63 ura3Δ0 YFL023W(BUD27)::kanMX4 YOR224C(RPB8)::ECFP::SpHIS5*	This work
YFN335	*MATa his3Δ1 leu2Δ0 met15Δ0 ura3Δ0 lys2Δ YOR224C(RPB8)::ECFP::SpHIS5*	This work
YFN348	*MATα his3Δ1 leu2Δ0 lys2Δ0 ura3Δ0 YJL179W(GIM6, PFD1)::kanMX4 YOR224C(RPB8)::ECFP::SpHIS5*	This work
YFN349	*MATα his3Δ1 leu2Δ0 lys2Δ0 ura3Δ0 YEL003W(GIM4, PFD2)::kanMX4 YOR224C(RPB8)::ECFP::SpHIS5*	This work
YFN350	*MATa his3Δ1 leu2Δ0 met15Δ0 ura3Δ0 YLR200W(YKE2, PFD6)::kanMX4 YOR224C(RPB8)::ECFP::SpHIS5*	This work
FY86	*MATα leu2Δ1 ura3-52 his3Δ200 RPB1-GFP::his5*	Gift from F. Estruch
YFN416	*MATa his3Δ1 leu2Δ0 met15Δ0 ura3Δ0 RPB1-GFP::his5*	This work
YFN417	*MATa his3Δ1 leu2Δ0 met15Δ0 ura3Δ0 trp1-Δ63 YFL023W(BUD27)::kanMX4 RPB1-GFP::his5*	This work
YFN418	*MATα his3Δ1 leu2Δ0 lys2Δ0 ura3Δ0 YJL179W(GIM6, PFD1)::kanMX4 RPB1-GFP::his5*	This work
YFN419	*MATα his3Δ1 leu2Δ0 lys2Δ0 ura3Δ0 YEL003W(GIM4,PFD2)::kanMX4 RPB1-GFP::his5*	This work
YFN420	*MATa his3Δ1 leu2Δ0 met15Δ0 ura3Δ0 YLR200W(YKE2, PFD6)::kanMX4 RPB1-GFP::his5*	This work

**Table 2:** Plasmids.

Name	Yeast markers	Origin
pCM189	ORI (CEN) *URA3*	[25]
pCM189-*BUD27*	ORI (CEN) *URA3*	This work
pCM189-*BUD27-GFP*	ORI (CEN) *URA3*	This work
pCM189-*BUD27ΔNES -GFP*	ORI (CEN) *URA3*	This work
pCM189-*BUD27-TAP*	ORI (CEN) *URA3*	This work
pCM189-*BUD27ΔPFD-TAP*	ORI (CEN) *URA3*	This work
pCM189-*BUD27ΔR5-TAP*	ORI (CEN) *URA3*	This work
pCM189-*BUD27ΔPFDR5-TAP*	ORI (CEN) *URA3*	This work
pFL44L	ORI (2 µm) *URA3*	[56]
pFL44L-*RPB5*	ORI (2 µm) *URA3*	[57]
pCM189-*IWR1ΔNES*	ORI (CEN) *LEU2*	Gómez-Navarro et al, submitted
pKT210		[48]

**Table 3:** Primers.

Name	Sequence
Rpb8ECFP-501	GAAATTTGAATAACTTGAAGCAAGAGAACGCTTATCTTTTGATTCGTCGTggtgacggtgctggttta
Rpb8ECFP-301	CACTTTTATAAAGTATTATTTATATTACTAGTAGCAGTAAGTGATCGCCC tcgatgaattcgagctcg
Rpb1-508	CCCAACATCTCCAGGCTACAGC
Rpb1-310	CGCAAGCCATGATTACTGG
hsURI1_02	GCCUGAUAAAUUGUCUUAUUU
hsURI1_02_as	AUAAGACAAUUUAUCAGGCUU
hsURI1E9-f01	ATTGACGACGATGATGGTGA
hsURI1E10-r01	GCCAGTGCTGTTCTTTCGTT
PPIA 501	TTCATCTGCACTGCCAAGAC
PPIA 301	TCGAGTTGTCCACAGTCAGC

### Protein tagging

Rpb8-ECFP and Rpb1-GFP tagging was performed by yeast recombination of a PCR fragment as described in Longtine *et al.* [47] amplified from plasmid pKT210 [48] or from chromosomal DNA from strain FY86, using Rpb8ECFP-501 and Rpb8ECFP-301 or Rpb1-308 and Rpb1-310 primers, respectively (see Table 3).

### Protein immunoprecipitation and TAP purification

400 ml cells growing exponentially (A_600_∼0.6–0.8) in yeast extract-peptone-dextrose (YPD) medium or synthetic minimal (SD) were washed twice with ultrapure water and lysis buffer (50 mM HEPES [pH 7.5], 120 mM NaCl, 1 mM EDTA, 0.3% Chaps 50%). Cells were resuspended in 1 ml lysis buffer supplemented with 1x protease inhibitor cocktail (Complete, Roche), 0.5 mM PMSF, 2 mM sodium orthovanadate and 1 mM sodium fluoride and whole-cell extracts were prepared using a MixerMill MM400 RETSCH (3 min 30 Hz). Immunoprecipitations were carried out as described elsewhere [49] with some modifications: 150 µl of whole-cell extract (2000 µg) and lysis buffer for all washes were used. 35 µl of Dynabeads M-280 Sheep anti-Mouse IgG (Invitrogen) were used with 9E10 anti C-Myc antibody (1 µg, Santa Cruz Biotechnology), 8WG16 anti-Rpb1 antibody (1.5 µg, Covance) and 12CA5 anti-HA antibodies (0.4 µg, ROCHE). For TAP purification, the same protocol was used with Dynabeads Pan Mouse IgG (Invitrogen). The affinity-purified proteins were released from the beads by boiling for 10 min. Eluted proteins were analysed by Western blot with different antibodies: 9E10 anti-C-Myc, 12CA5 anti-HA, 8WG16 anti-Rpb1, PAP, anti-POLR2C (1Y26, Abcam), and anti-Rpb6 or anti-Rpb5 (a gift from M. Werner).

TAP purification for protein identification by mass spectrometry was performed as described previously [50]. Bud27-TAP fusion protein and associated proteins were recovered from cell extracts by affinity selection on an IgG matrix. After washing, the TEV protease is added to release the bound material. The eluate is incubated with calmodulin-coated beads in the presence of calcium. After washing, the bound material is released with EGTA. This enriched final fraction was analyzed by mass spectrometry using the MudPIT approach as described in [35].

### Immunolocalization and fluorescence microscopy

Cells were grown at 30°C in YPD or SD medium (A_600_∼0.5–0.7), fixed with 37% w/v formaldehyde at room temperature for 2 h with slow shaking, and then centrifuged and washed twice with PBS. Cells were resuspended in spheroplasting buffer (1.2 M sorbitol, 0.1 M K-phosphate buffer pH 6.5) and cell wall digested with 125 µg/ml zymolyase 20T (USBiological) and 22.7 mM 2-mercaptoethanol (SIGMA) by incubation for 1 h at 37°C without shaking. The spheroplasts were washed twice with PBST (PBS with 0.05% Tween 20) and then resuspended in the same solution. Cell suspension was added to an AAS (3-aminopropyltriethoxysilane, Sigma) slide, incubated at room temperature until slide was dry and washed twice with PBST. Then, 50 µl of PBS-BSA (1 mg/ml BSA) were added, and the slides. After incubation for 30 min in a humid chamber were washed three times with PBS. Next, 50 µl of 1∶100 dilution of the primary antibodies (8WG16, anti C-Myc or anti-HA) in PBS-BSA (1 mg/ml BSA) were added and incubated 2 h at room temperature in a humid chamber. Slides were then washed three times with PBS, and incubated for 1 h, in the dark, at room temperature in a humid chamber with 50 µl of 1∶100 dilution of secondary antibody (Cy2 antimouse; Jackson Labs). The slides were washed three times with PBS and incubated for 5 min with 50 µl of 1 µg/ml DAPI (in PBS). After washing three times with PBS, slides were finally covered with a Vectashield (Vector Laboratories) mounting solution.

Human cells were fixed for 15 min at room temperature with 4% (v/v) paraformaldehyde in PBS. Following fixation, cells were washed three times for 15 min with PBS, treated with 50 mM ammonium chloride for 30 min and permeabilised for 20 min with PBS containing 0.1% (v/v) Triton X-100 (wash solution). Blocking solution (wash solution with 5% (w/v) BSA) was then added for 30 min and then, cells incubated for 1 h with 1∶100 dilution of the primary antibodies (8WG16) in blocking solution. Cells were washed three times for 15 min with wash solution and then incubated with 1∶100 dilution of secondary antibody (Cy2 antimouse; Jackson Labs) for 1 h in blocking solution. After washing three times with wash solution, 15 min each, slides were finally covered with a Vectashield (Vector Laboratories) mounting solution containing DAPI.

The fluorescence intensity was scored with a fluorescence microscope (Olympus BX51).

### Chromatin isolation

Chromatin isolation was performed as previously described [51] with some modifications. Briefly, about 5×10^8^ cells growing exponentially (A_600_∼0.6–0.8) were resuspended in 3 ml of 100 mM PIPES/KOH (pH 9.4) containing 10 mM DTT and 0.1% sodium azide and then incubated at room temperature for 10 min. Cells were spun down, resuspended in 2 ml of 50 mM phosphate buffer (pH 7.5), containing 0.6 M Sorbitol, 10 mM DTT, and 4 µl of 20 mg/ml zymoliase and incubated 10 min at 37°C in a water bath to spheroplast formation. Spheroplasts were then pelleted at 4°C, washed with 50 mM HEPES-HOK buffer (pH 7.5) containing 100 mM KCl, 2.5 mM MgCl_2_ and 0.4 M Sorbitol, resuspended in equal volume (∼80 µl) of EBX buffer (50 mM HEPES/KOH (pH 7.5), 100 mM KCl, 2.5 mM MgCl_2_, 0.25% Tritón-X100, 0.5 mM PMSF, 0.5 mM DTT, cocktail protease inhibitors *Complete Roche* 1x) and incubated for 3 min on ice. This whole cell extract was laid onto 400 µl of EBX-S buffer (EBX with sucrose 30%) and centrifuged at 12000 rpm for 10 min. After the sucrose gradient a chromatin pellet became visible and was washed with 400 µl of EBX buffer and finally resuspended in 100 µl of the same solution. A 1/10 dilution of chromatin pellet was used for SDS-PAGE and Western blotted with antibodies against Rpb1 (8WG16), alfa-tubulin (T5168; Sigma-Aldrich) and Nop1 (28F2; Abcam).

### RNA silencing (siRNA)

URI, prefoldin-like chaperone (Gene ID: 8725) gene was silenced by transfection with the siRNA heteroduplex hsURI1_02 and hsURI1_02_as (Table S1). Human pulmonary fibroblast (HPF) were cultured in growth medium (GM), consisting of DMEM supplemented with 10% fetal bovine serum, 2 mM l-glutamine, and 50 U/ml penicillin–streptomycin. Cells were seeded in twenty four-well plates (30,000 cells per well) and transfected in triplicate with 40–200 nM heteroduplex oligonucleotides using the Lipofectamine 2000 Transfection Reagent (Invitrogen), following the manufacturer's protocol, and incubated for 24 h at 37°C. Control cells were treated in the same conditions without siRNA heteroduplex. The experiments were performed three times (three replicates). After indicated time, cells were either harvested for RNA extraction or fixed with PFA 4% in PBS for inmunolocalisation analysis.

### RNA isolation and quantitative real-time PCR (q–RT–PCR)

Total RNA was isolated from URI silenced HPF cells using the SV Total RNA Isolation System (Promega), according to the manufacturers. Retrotranscription was performed from 200 ng of total RNA using the Maxima First Strand cDNA Synthesis Kit (Fermentas) in a final volume of 20 µl, according to the manufacturer's protocol. As a control, each sample was subjected to the same process without reverse transcriptase.

URI mRNA accumulation was analyzed by q-RT-PCR with oligonucleotides hsURI1E9-f01 and hsURI1E10-r01 using cDNA corresponding to 10 ng. Human cyclophilin A (PPIA) was used as an internal control. Each PCR reaction was performed at least three times, with three independent samples. All oligonucleotides used are indicated in Table 3.

## Supporting Information

Figure S1 Δ*bud27 mutant* phenotypes are corrected by overexpression of different *BUD27* constructions. Growth of wild-type and Δ*bud27* mutant strains transformed with different constructions containing whole *BUD27* or deleted forms of *BUD27*, at different temperatures; *pCM* and *pFL* correspond to the control empty vectors.
(TIF)

Figure S2 RNA pols localisation. A) Deletion of Bud27 NES domain does not impair RNA pols nuclear localisation (Rpb8-ECFP). Δ*bud27* mutant cells overexpressing Bud27ΔNES-GFP were grown at 30°C and localisation of Bud27ΔNES-GFP and Rpb8-ECFP was analysed *in vi*vo. B) *BUD27* overexpression (*pCM-BUD27-TAP*) corrects nuclear localization of Rpb8-ECFP in Δ*bud27* mutant cells at 30°C when compared to the same strain containing an empty plasmid (*pCM*).
(TIF)

Figure S3 siRNA silencing of URI. URI mRNA accumulation was determined in human pulmonary fibroblast grown in GM medium at 37°C transfected with 40, 100 and 200 nM of siRNA(URI) heteroduplex. CONTROL cells were treated in the same conditions without siRNA heteroduplex.
(TIF)

Figure S4 *RPB5* overexpression rescues nuclear RNA pols localisation. Immunocytochemistry experiments using antibodies against Rpa190-HA (anti-HA), Rpb1 (8WG16), and Rpc160-Myc (anti-Myc) in Δ*bud27* mutant cells with tagged Rpa190-HA (RNA pol I) and Rpc160-Myc (RNA pol III), at 30°C, transformed with a plasmid overexpressing *RPB5* (*pFL-RPB5*), or with an empty vector (*pFL*).
(TIF)

Table S1 Rpb3-TAP purification from wild type and Δ*bud27* mutant containing functional tagged version of Rpb3 (Rpb3-TAP). The protein mixture obtained in each case was subjected to multidimensional protein identification technology (MudPIT) [35] and their ratios versus Rpb3 calculated.
(DOC)
