# Supplementary figures and images for: The Prefoldin Bud27 Mediates the Assembly of the Eukaryotic RNA Polymerases in an Rpb5-Dependent Manner
Source: PLoS Genet. 2013 Feb 14;9(2):e1003297. doi: 10.1371/journal.pgen.1003297 (PMC3573130; doi:10.1371/journal.pgen.1003297)

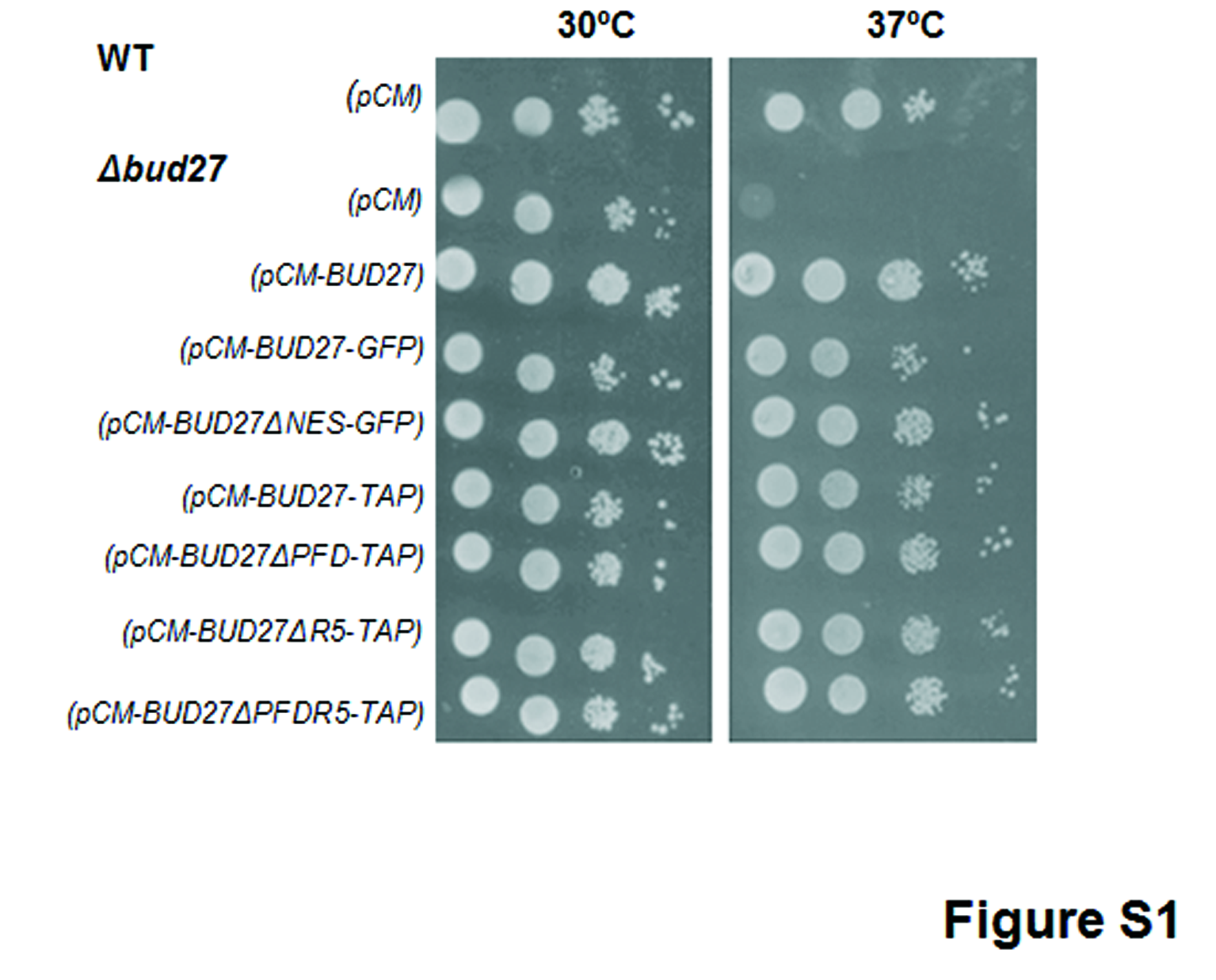

Supplement: Figure S1 — Δbud27 mutant phenotypes are corrected by overexpression of different BUD27 constructions. Growth of wild-type and Δbud27 mutant strains transformed with different constructions containing whole BUD27 or deleted forms of BUD27, at different temperatures; pCM and pFL correspond to the control empty vectors. (TIF) [file pgen.1003297.s001.tif]

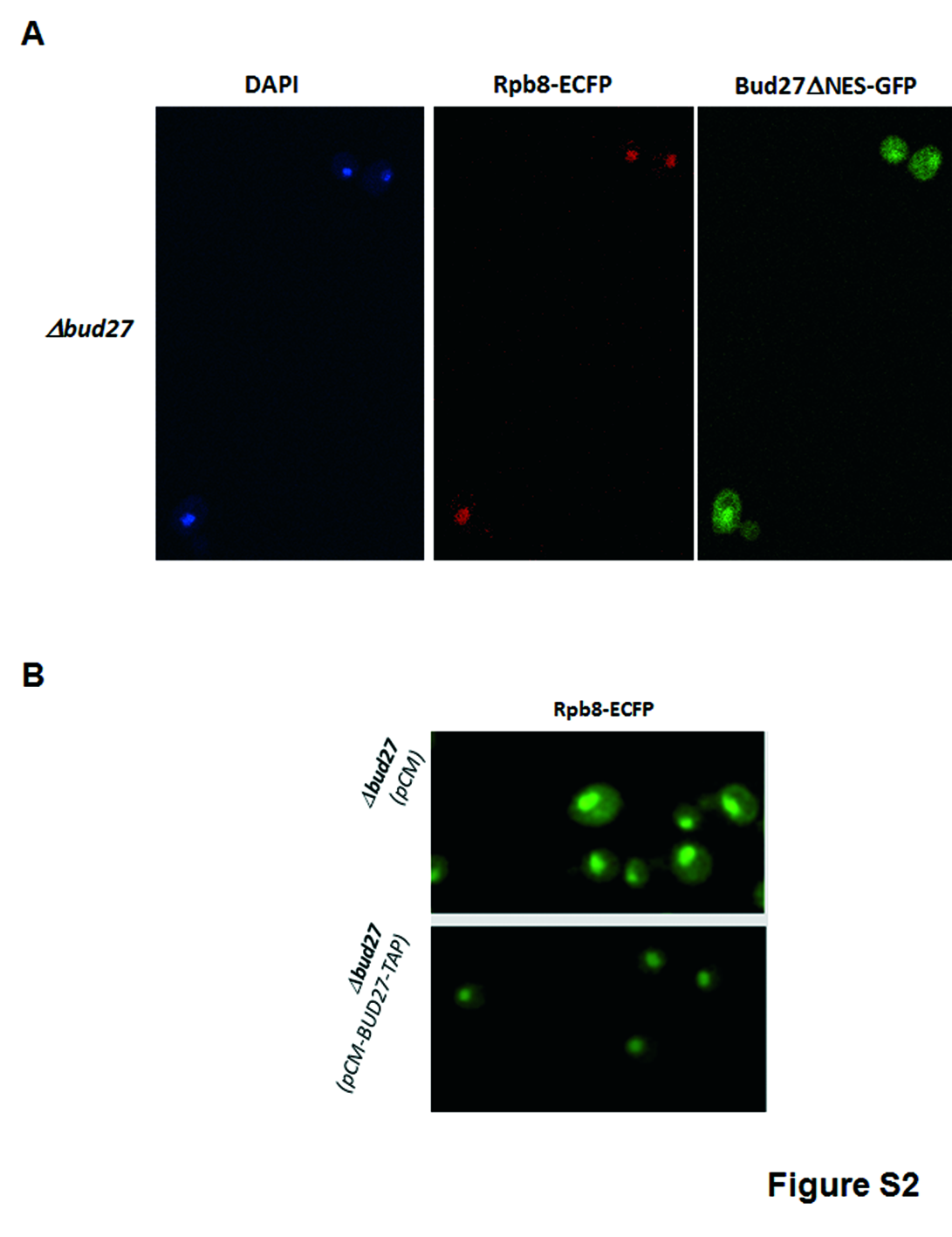

Supplement: Figure S2 — RNA pols localisation. A) Deletion of Bud27 NES domain does not impair RNA pols nuclear localisation (Rpb8-ECFP). Δbud27 mutant cells overexpressing Bud27ΔNES-GFP were grown at 30°C and localisation of Bud27ΔNES-GFP and Rpb8-ECFP was analysed in vivo. B) BUD27 overexpression (pCM-BUD27-TAP) corrects nuclear localization of Rpb8-ECFP in Δbud27 mutant cells at 30°C when compared to the same strain containing an empty plasmid (pCM). (TIF) [file pgen.1003297.s002.tif]

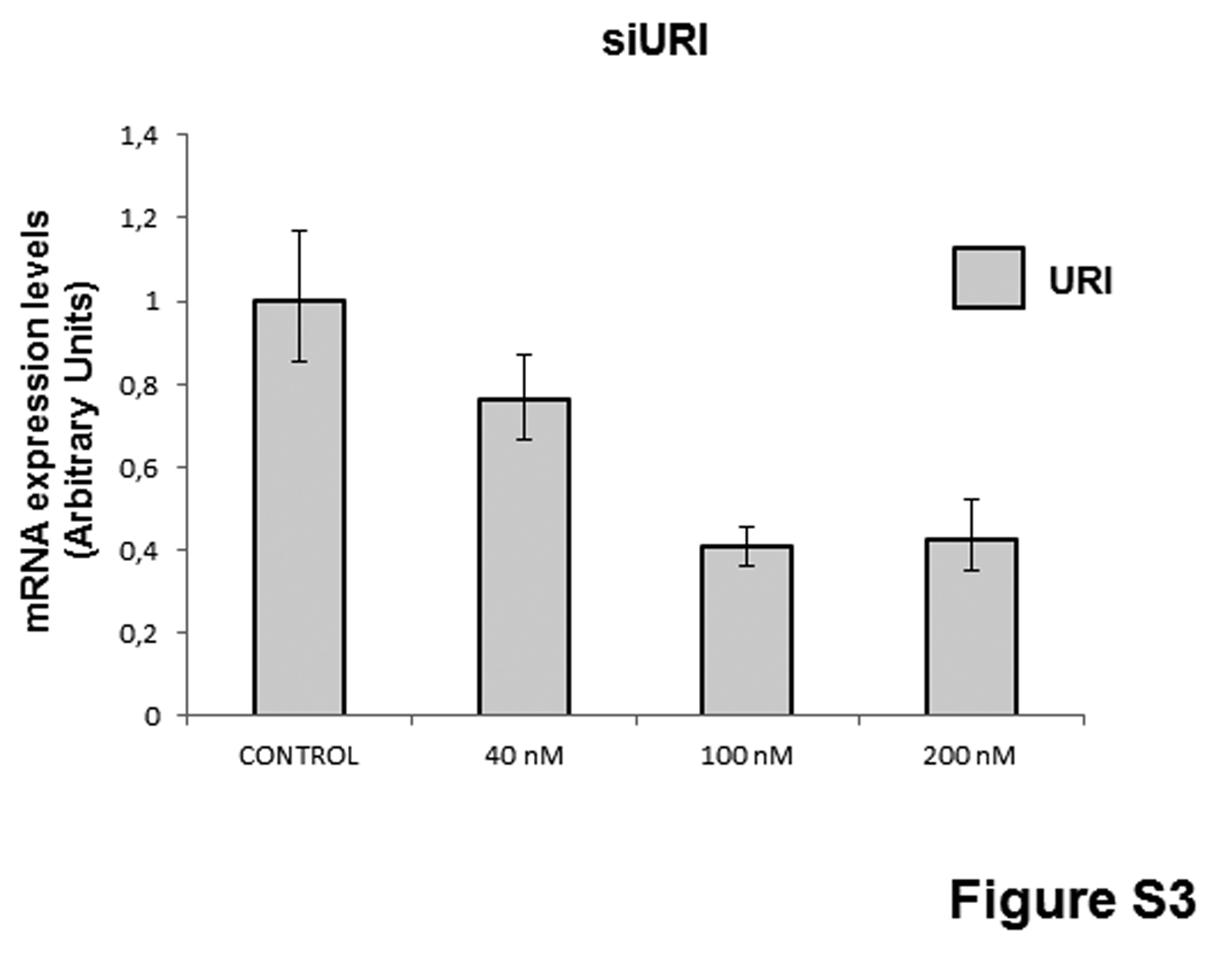

Supplement: Figure S3 — siRNA silencing of URI. URI mRNA accumulation was determined in human pulmonary fibroblast grown in GM medium at 37°C transfected with 40, 100 and 200 nM of siRNA(URI) heteroduplex. CONTROL cells were treated in the same conditions without siRNA heteroduplex. (TIF) [file pgen.1003297.s003.tif]

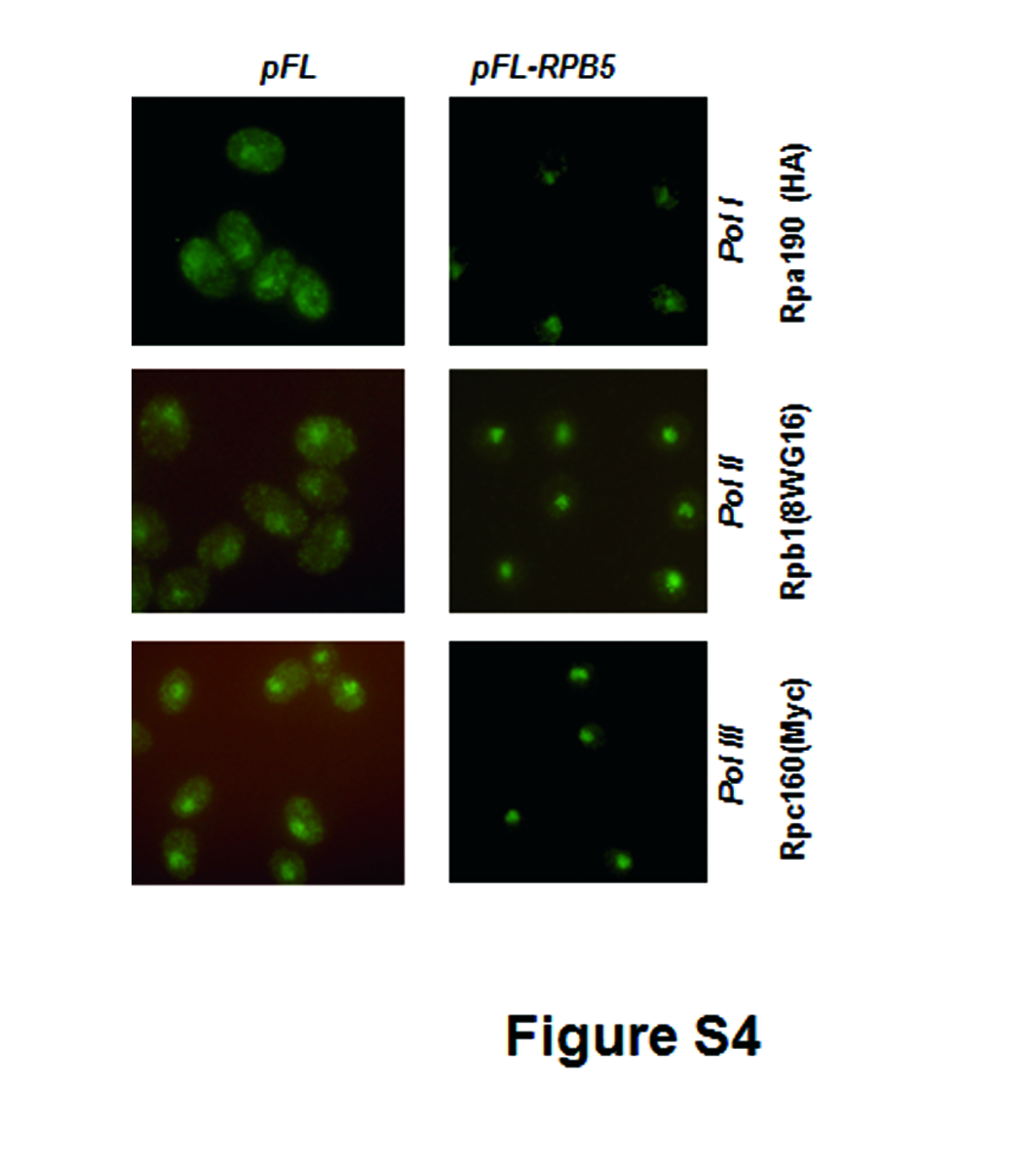

Supplement: Figure S4 — RPB5 overexpression rescues nuclear RNA pols localisation. Immunocytochemistry experiments using antibodies against Rpa190-HA (anti-HA), Rpb1 (8WG16), and Rpc160-Myc (anti-Myc) in Δbud27 mutant cells with tagged Rpa190-HA (RNA pol I) and Rpc160-Myc (RNA pol III), at 30°C, transformed with a plasmid overexpressing RPB5 (pFL-RPB5), or with an empty vector (pFL). (TIF) [file pgen.1003297.s004.tif]
