# Supplementary material for: The Prefoldin Bud27 Mediates the Assembly of the Eukaryotic RNA Polymerases in an Rpb5-Dependent Manner
Source: PLoS Genet. 2013 Feb 14;9(2):e1003297. doi: 10.1371/journal.pgen.1003297 (PMC3573130; doi:10.1371/journal.pgen.1003297)
Supplement: Table S1 — Rpb3-TAP purification from wild type and Δbud27 mutant containing functional tagged version of Rpb3 (Rpb3-TAP). The protein mixture obtained in each case was subjected to multidimensional protein identification technology (MudPIT) [35] and their ratios versus Rpb3 calculated. (DOC) [file pgen.1003297.s005.doc]

**TABLE S1**

| **Rpb3 ratios** |  |  |
| --- | --- | --- |
|  |  |  |
|  | **Rbp3TAP** | **Rpb3TAP *bud27Δ*** |
| **Rpb1** | 1,10610998 | 0,30210315 |
| **Rpb2** | 2,0586558 | 0,95645741 |
| **Rpb3** | 1 | 1 |
| **Rpb4** | 166,796538 | 16,1336927 |
| **Rpb5** | 0,33645621 | 0,0584443 |
| **Rpb6** | 2209,92281 | 203,002484 |
| **Rpb7** | 0,02505092 | 0,01384485 |
| **Rpb8** | 0,03095723 | 0,00803213 |
| **Rpb9** | 0,6712831 | 0,14056225 |
| **Rpb10** | 13,9167006 | 7,18399915 |
| **Rpb11** | 0,88431772 | 0,56785035 |
| **Rpb12** | 1,60590631 | 0,28762418 |
